# Do statins benefit low-risk population for primary prevention of atherosclerotic cardiovascular disease: A retrospective cohort study

**DOI:** 10.3389/fmed.2022.1024780

**Published:** 2022-11-03

**Authors:** In Sun Ryou, Ju Young Kim, Hwa Yeon Park, Sohee Oh, Sehun Kim, Hwa Jung Kim

**Affiliations:** ^1^Department of Family Medicine, Ewha Womans University Medical Center, Ewha Womans University School of Medicine, Seoul, South Korea; ^2^Department of Family Medicine, Seoul National University Bundang Hospital, Seoul National University College of Medicine, Seoul, South Korea; ^3^Department of Biostatistics, Seoul Metropolitan Government Seoul National University Boramae Medical Center, Seoul, South Korea; ^4^Cardiovascular Center, Hallym University Medical Center, Seoul, South Korea; ^5^Department of Preventive Medicine, Ulsan University College of Medicine, Seoul, South Korea; ^6^Department of Clinical Epidemiology and Biostatistics, ASAN Medical Center, Seoul, South Korea

**Keywords:** atherosclerotic cardiovascular disease, statin, primary prevention, major adverse cardiovascular disease, low-density lipoprotein cholesterol

## Abstract

The reported beneficial effects of statins on cardiovascular outcome based on risk assessment are inconsistent. Therefore, we investigated statin therapy effectiveness for the primary prevention of cardiovascular disease (CVD), according to the Korean Risk Prediction Model (KRPM). Subjects aged 40–79 years with low density lipoprotein cholesterol (LDL-C) of < 190 mg/dL and without CVD history were categorized as statin users or non-users using the National Health Insurance Service-National Sample Cohort (NHIS-NSC) database, Korea, 2002–2015. The 10-year atherosclerotic CVD (ASCVD) risk was calculated using the validated KRPM and categorized as low, borderline, intermediate, or high-risk groups; the incidence of major adverse cardiovascular events (MACEs) was compared over a mean follow-up period of 5.7 years using Cox proportional hazard models. The MACE incidence risk was decreased in statin users [hazard ratio (HR) 0.90, 95% confidence interval (CI) (0.84–0.98)]. However, there was an increased risk of MACE incidence in low-risk statin users [HR 1.80, 95% CI (1.29–2.52)], and no significant relationship was identified between statin use and MACE in the borderline [HR 1.15, 95% CI (0.86–1.54)] and intermediate-risk [HR 0.94, 95% CI (0.85–1.03)] groups. The risk of MACE incidence decreased only in the high CVD risk group among statin users. Statin use is not associated with MACE reduction in low- to intermediate-risk participants. Therefore, individuals with LDL-C level of < 190 mg/dL and low ASCVD risk should consider statin therapy only when CVD risk is proved obvious using an appropriate ASCVD risk tool.

## Introduction

Cardiovascular disease (CVD) is the leading cause of death worldwide (1, 2). The National Statistical Office proclaimed CVD as the second leading cause of death in Korea, with the number of affected patients increasing from 44.1 to 60.2 per 100,000 people between 2007 and 2017 (3).

Recent guidelines recommend statin therapy to reduce CVD, depending on the patient's low-density lipoprotein cholesterol (LDL-C) levels (lipid-based) as well as the estimated risk of CVD (risk-based) (4–9). Most guidelines have suggested that statin therapy is beneficial when LDL-C is ≥190 mg/dL. The West of Scotland Coronary Prevention Study (WOSCOPS) study has reported that statin therapy reduced atherosclerotic CVD (ASCVD) when the LDL-C was ≥190 mg/dL, regardless of risk (10). According to the 2019 European Society of Cardiology/European Atherosclerosis Society (ESC/EAS) guidelines for the management of dyslipidemia, statin therapy is effective in cases of high single risk factors such as an LDL-C level of >190 mg/dL (11). The 2019 American College of Cardiology/American Heart Association (ACC/AHA) Guidelines on the Primary Prevention of Cardiovascular Disease recommended statin therapy for patients with LDL-C of >190 mg/dL as first-line treatment for the primary prevention of ASCVD without the need for risk assessment (4, 7). These guidelines consider the benefits of statin therapy to outweigh the risk of adverse effects in patients with LDL-C level of ≥190 mg/dL (4, 7, 10, 11). However, with respect to risk-based assessment, the CVD risk estimates and eligibility for receiving statins differ somewhat between several guidelines. The 2019 ESC/EAS guidelines have used the European SCORE (Systematic Coronary Risk Estimation) system to calculate the 10-year risk of fatal CVD based on the following risk factors: age, gender, smoking, systolic blood pressure, and total cholesterol. In addition, they have recommended statin therapy based on cardiovascular risk. For primary prevention, individuals with very high risk (patients with diabetes with end-organ damage, three major risk factors, severe chronic kidney disease [CKD; estimated glomerular filtration rate {eGFR} < 30], heterozygous familial hypercholesterolemia with another major risk factor, or a SCORE ≥10%) or high risk (total cholesterol >310 mg/dL, LDL-C >190 mg/dL, BP 180/110 mm Hg, diabetes for >10 years or with one major risk factor, moderate CKD [eGFR 30–59], or SCORE 5–9%) are recommended for statin therapy. The US Preventive Services Task Force has announced that the use of statins for the primary prevention of CVD is beneficial in adults between the ages of 40 and 75 years, without a history of CVD, who present one or more CVD risk factors (dyslipidemia, diabetes mellitus (DM), hypertension, and smoking), and an estimated 10-year CVD risk ≥10% (5).

The latest ACC/AHA guidelines for blood cholesterol management recommend statin therapy as the primary approach to prevent CVD in individuals aged 40–75 years with DM and LDL-C levels between 70 and 190 mg/dL; or individuals aged 40–75 years without DM and with LDL-C levels ≥70–189 mg/dL at a 10-year ASCVD risk of ≥7.5–19.9% (4). Furthermore, these guidelines suggested that 10-year ASCVD risk estimates should be reclassified based on individual risk-enhancing clinical factors, including individual socioeconomic status or medical accessibility, and specific racial/ethnics groups. The guidelines further recommended coronary artery calcium measurement to refine the risk assessment for individuals belonging to the borderline- (5% to < 7.5%) and intermediate- (≥7.5% to < 20%) risk groups, for whom the uncertainty remains regarding statin effectiveness remains (7).

To establish a more accurate eligibility criteria for statin use based on risk, we excluded individuals with LDL-C level of ≥190 mg/dL in which the effect of statin has been previously reported. Additionally, we considered not only individual risk but also population risk including race and baseline CVD prevalence or incidence as the absolute risk reduction and net benefit from statin therapy based on the background population risk. Even for those with similar CVD risk profiles, the number needed to be treated for preventing one ASCVD event is much higher for people in countries with low ischemic mortality (12). A recent systematic review suggested that the efficacy of statins in primary prevention is limited and largely depends on the baseline risk (13). Thus, it is imperative to identify individuals who are eligible for statin therapy for primary prevention based on accurate estimates of CVD risk scores for target populations.

In this study, we used a validated Korean Prediction Risk Model specific to Koreans, that uses the 2013 ACC/AHA pooled cohort equations (PCE) by recalibrating the coefficient derived from the Korean Heart Study (KHS) data to determine whether statin use has a CVD-protective effect even in a relatively low CVD risk race group, especially where the effect of statin use is ambiguous.

## Methods

### Data source

Most Koreans are enrolled with the National Health Insurance (NHI), a non-profit organization and single insurer that manages the National Health Insurance Service (NHIS). The NHI manages enrolled and insured individuals and their dependents, collects contributions, and sets medical fee schedules. It also provides biennial health check-ups free of charge for Koreans aged over 20 years enrolled in the NHI. To process these tasks, the NHIS constructed a data warehouse to collect information on insurance eligibility, insurance contributions, medical history, and medical institutions. This system enables the NHIS to maintain national records for healthcare service utilization and prescriptions (14).

Accordingly, the NHIS has formed a National Health Information Database that provides researchers with two types of databases. The Sample Research Database refers to standardized data set in a sharable form by extracting Korean samples and is encrypted so that a specific person cannot be recognized in the data. However, the database allows for long-term observation as a cohort on the same encrypted individual through connections in the qualification data, including social and economic variables (location of residence, month and date of death, cause of death, and income rank), treatment details, and medical check-up data. There are five data sets, namely, the National Sample Cohort (NSC), medical check-up, elderly cohort, working women cohort, and infant medical check-up databases (14, 15).

The highly representative NHIS-NSC database, comprising one million Koreans qualified to receive health insurance and medical benefits for 1 year in 2006 who were followed up from 2002 to 2015, was considered in this study. The NHIS-NSC data include health screening laboratory results, socioeconomic status, claims for prescription medication, diagnosis codes, type of hospital visit, length of stay, and date and cause of death (14, 15).

### Ethics approval

The Institutional Review Board of Seoul National University Bundang Hospital (IRB of SNUBH) approved the current study (approval number: X-1611-372-905). The need for patient consent was waived by the Ethics Committee of the IRB of SNUBH owing to the retrospective nature of the study and the strict anonymization of data. All methods were conducted in accordance with the ethical standards of the 1964 Declaration of Helsinki and its later amendments, or comparable ethical standards.

### Study cohort construction

**Figure 1:**
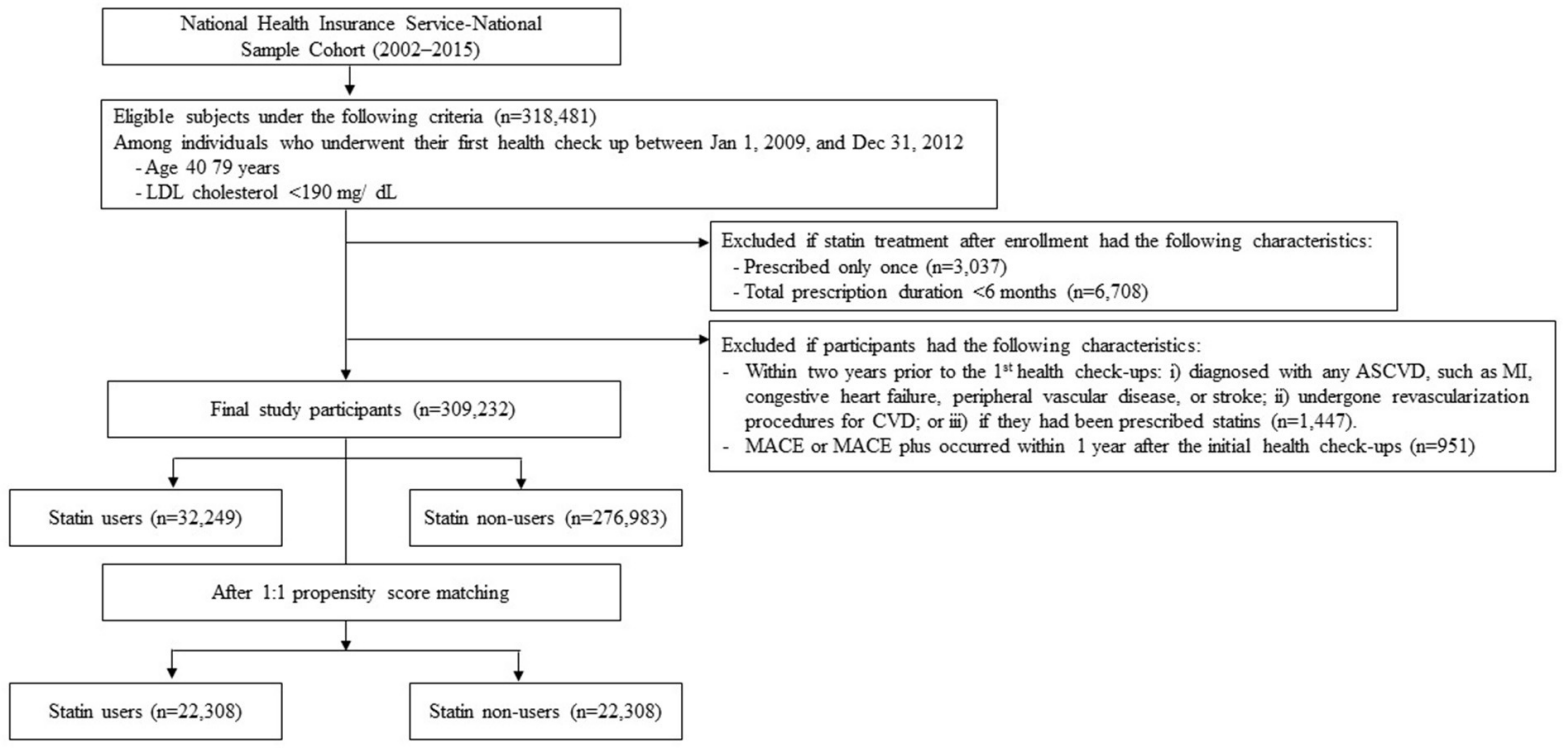
Flow chart of study participant selection. An illustration of the final study population selection, summarizing the inclusion and exclusion criteria in this retrospective cohort study.

Using NHIS-NSC data, participants aged between 40 and 79 years with an LDL-C level < 190 mg/dL and who had their first health check-ups between January 1, 2009 and December 31, 2012 were selected. The date collected during the first health check-up was defined as the cohort entry date. Patient data were excluded if the respective patients had died within 1 year from the cohort entry date. Patients were excluded if they fulfilled the following criteria within two years prior to the cohort entry date: (i) diagnosed with any ASCVD, such as myocardial infarction (MI), congestive heart failure, peripheral vascular disease, or ischemic stroke, listed in the International Classification of Diseases 10th Revision (ICD-10); (ii) undergone revascularization procedures for CVD; or (iii) had been prescribed statins (Supplementary Table S1). The study population was divided according to their medical prescription records into two groups, namely, statin users and non-users (Figure 1).

### Definition of statin users and index date

We adopted a new user design, and participants were classified as statin users (simvastatin, pravastatin, lovastatin, fluvastatin, rosuvastatin, atorvastatin, or pitavastatin) if they had more than two records of statin prescription within 2 years after the cohort entry date, and if the total duration of the prescriptions was longer than 6 months. We excluded the users with late statin prescription, i.e., those who were prescribed statins after 2 years from the cohort entry date to avoid misclassification bias.

**Figure 2:**
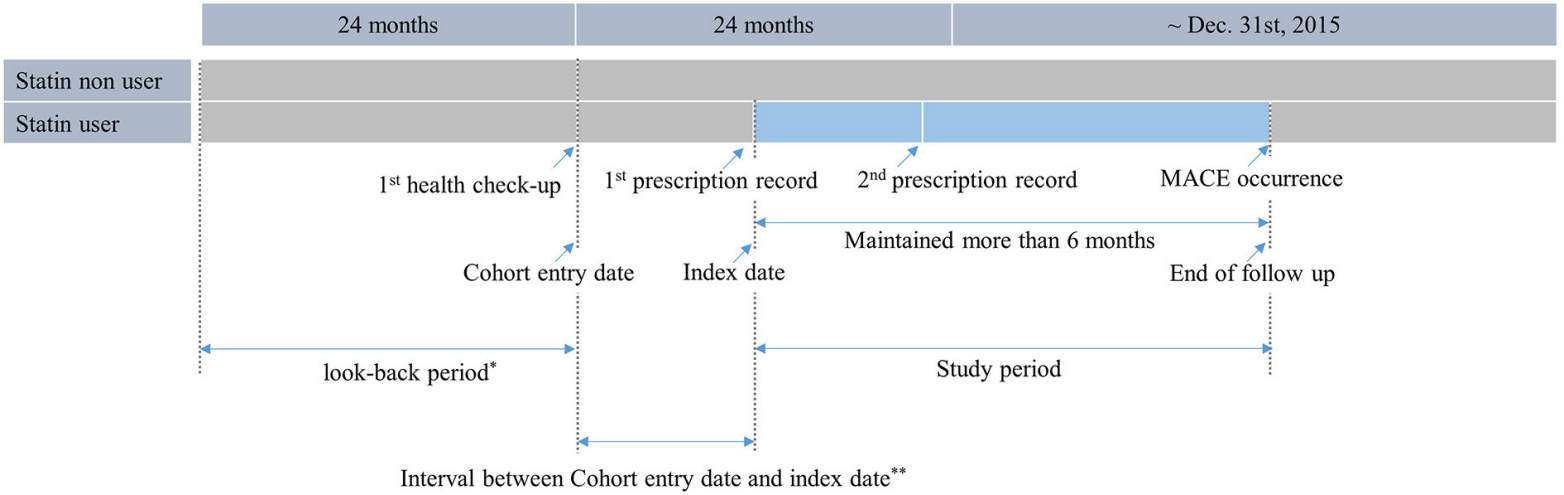
Operational definition of statin users. Statin users were defined as those who received health check-up between 2009 and 2012, had a record of being prescribed statins at least twice within 2 years after the first health check-up, and were prescribed statins for more than 6 months.

The total duration of statin treatment was determined by combining all prescription periods using the claims database. Participants who did not receive statins during the study period were identified as non-users. Among statin users, the index date was defined as the date of the first statin prescription. For statin non-users, a proxy index date was assigned based on the distribution of intervals between the cohort entry date and index date of statin users (Proxy index date = cohort entry date of statin non-users + interval between the cohort entry date and index date of statin users). The index dates of non-users were randomly selected according to the distribution of the index dates of statin users (Figure 2) (16). See Appendix B for more information on the sample data and programs used.

### Risk categories using Korean-specific 10-year ASCVD risk

To calculate the 10-year ASCVD risk for Korean adults, the 2013 ACC/AHA PCE were adopted and recalibrated based on the derivations from the KHS dataset (17). The KHS included 430,920 individuals (266,782 men and 164,138 women), who had voluntarily undergone health screening and had no previous CVD history, in 18 centers across South Korea between 1996 and 2004. (18) Using this population, Jee et al. developed a Korean-specific pooled equation for ASCVD risk adopting the method proposed by D'Agostino et al. (19, 20). In the recalibrated equation, the coefficients were determined from the Cox model of the 2013 ACC/AHA equation, and the mean values for risk factors and baseline survival rates were replaced by values from the KHS cohort data (21). As shown in Supplementary A, the equation for the Korean Risk Prediction (KRPM) Model for ASCVD was developed. This equation was used to calculate the Korean-specific 10-year ASCVD risk for the population and proved effective in distinguishing cases from non-cases (area under the receiver operating characteristic curve: 0.741 for men [95% confidence interval [CI], 0.732–0.750), 0.745 for women (95% CI, 0.734–0.757)], while also demonstrating good predictive value for CVD events [Hosmer-Lemeshow χ^2^: 25.90 for men (*P* = 0.002), 14.69 for women (*P* = 0.100)] (18). The predicted CVD risks were categorized into four groups according to the score: low-risk (< 5% 10-year ASCVD risk), borderline-risk (5 to < 7.5% 10-year ASCVD risk), intermediate-risk (≥7.5 to < 20% 10-year ASCVD risk) and high-risk (≥20% 10-year ASCVD risk).

### Ascertainment of baseline covariates

At the first health check-up, demographic information including age, sex, income, and residential area were collected. Clinical characteristics such as systolic blood pressure (mmHg), diastolic blood pressure (mmHg), body mass index (BMI; kg/m^2^), waist circumference (cm), total cholesterol (mg/dL), triglyceride (mg/dL), high-density lipoprotein-cholesterol (mg/dL), LDL-C (mg/dL), fasting blood glucose (mg/dL), glomerular filtration rate (mg/dL), aspartate aminotransferase (IU/L), alanine aminotransferase (IU/L), and gamma-glutamyl transferase (IU/L) were also collected. History of comorbid diseases such as hypertension or diabetes, drug history, and lifestyle factors, such as smoking (never, former, current), alcohol consumption (excessive drinking: ≥4 standard drinks per day), and exercise (regular exercise: more than 20 min at a time at least three times a week) were determined through a questionnaire conducted at the first health check-up. We also considered the underlying comorbidity of subjects two years prior to the index date using the Modified Charlson Comorbidity Index with ICD-10 codes (22).

### Propensity score matching

To reduce selection bias, as well as potential confounders, we applied a 1:1 propensity score (PS) matching using a logistic regression method to associate statin use with baseline covariates. We calculated the standardized difference (defined as the difference in means or proportions divided by the standard error) and imbalance (defined as an absolute value > 0.20–small effect size) between the two groups for both continuous and categorical variables (23).

### Determination of outcomes and follow-up

The primary outcome was MACEs including MI, ischemic stroke, and cardiovascular death. To further consolidate the results, MACE plus, an extended range of MACE, was confirmed. MACE plus comprised of transient ischemic attack (TIA), unstable angina, and coronary revascularization. These outcomes were defined as when a participant was admitted to a hospital, visited an emergency department with the primary ICD-10 diagnostic code for the above conditions, or underwent revascularization procedures resulting from the above conditions.

To evaluate the relationship between statins and MACE, follow-up continued from the index date to the date when the first MACE was diagnosed, or December 31, 2015, whichever came first. We then followed up the patients until the occurrence of MACE plus—or December 31, 2015—to explore the relationship between statins and MACE plus. We excluded cases in which MACE or MACE plus occurred within 1 year of enrollment to avoid the possible effects of unmeasured, pre-existing risks.

### Statistical analysis

All data are represented as mean ± standard error for continuous variables (non-normal distribution is represented by median/interquartile range) and as Frequencies and percentages for categorical variables. We estimated the hazard ratios (HRs) of MACE and MACE plus according to statin use using a Cox proportional hazard model. Additionally, we compared the effect of statin use on MACE and MACE plus depending on the revised 10-year ASCVD risk.

In the subgroup analysis, we confirmed the effect of statin use on MACE and MACE plus depending on sex, age, history of hypertension or DM, and LDL-C levels.

Furthermore, the incidence and HR of new-onset DM were evaluated based on statin use in PS-matched cohort individuals without a history of DM diagnosis or drug use. New-onset DM was defined as the condition when the subjects had a diagnostic code for DM (ICD-10 E10–E14), fasting blood glucose level ≥126 mg/dL, or had been prescribed hypoglycemic drug (ATC code A10B) or insulin (ATC code A10A).

Statistical analyses were carried out using SAS Version 9.4 statistical software (Cary, NC, USA) and results were considered significant at *p* < 0.05.

## Results

### Baseline characteristics between statin users and non-users

Between 2009 and 2012, 309,232 subjects with LDL-C levels < 190 mg/dL, aged 40 to 75 years and who had no history of ASCVD or revascularization procedure for CVD or a record of statin prescription were included.

Statin users (*n* = 32,242) and non-users (*n* = 276,983) were identified. Baseline sociodemographic, clinical, and health behavioral factors are shown in Table 1. On average, statin users were older (59.59 vs. 52.97), had a higher BMI (25.19 vs. 23.80), and showed a higher prevalence of hypertension (77.52% vs. 27.61%) and diabetes (49.31% vs. 15.05%) than non-users. After performing 1:1 PS matching, PS-matched statin users (*n* = 22,308) and non-users (*n* = 22,308) were identified.

**Table 1:** Baseline characteristics of the study population.

	**Original cohort**					**PS matched cohort**				
	**Statin non-users**		**Statin users**		**SE/**	**Statin non-users**		**Statin users**		**SE/**
	**(*****n*** = **276,983)**		**(*****n*** = **32,249)**		***P-*value**	**(*****n*** = **22,308)**		**(*****n*** = **22,308)**		***P* value**
Age (years), SD	52.97	9.90	59.59	9.03	0.0577	59.98	10.67	59.52	8.86	0.0928
Sex *(N)*, %					< 0.0001					0.0025
Male	132,170	47.72%	12,863	39.89%		9,432	42.28%	9,117	40.87%	
Female	144,813	52.28%	19,386	60.11%		12,816	57.72%	13,191	59.13%	
Systolic blood pressure (mmHg), SD	123.10	15.42	128.80	15.75	0.0909	129.10	16.00	128.60	15.68	0.1500
Diastolic blood pressure (mmHg), SD	76.68	10.28	78.89	10.27	0.0605	79.20	10.21	78.83	10.21	0.9067
Body mass index (kg/m^2^), SD	23.80	6.69	25.19	3.17	0.0378	25.10	21.21	25.10	3.09	0.1435
Waist circumference (cm), SD	80.41	8.72	84.43	8.59	0.0512	83.83	8.66	84.23	8.43	0.0809
Total cholesterol (mg/dL), SD	196.60	32.78	198.70	43.14	0.2001	201.70	33.85	200.00	43.01	0.3665
^*^Triglyceride (mg/dL), IQR	109	76–161	135	96–195	0.5879	134.5	91–195	135	97–195	1.1155
^*^High-density lipoprotein cholesterol (mg/dL), IQR	53	45–63	52	45–62	0.158	52	44–62	53	45–62	0.3497
Low-density lipoprotein cholesterol (mg/dL), SD	115.20	30.24	112.20	38.83	0.1838	116.40	31.86	113.40	38.90	0.3367
^*^Fasting blood glucose (mg/dL), IQR	94	87–103	101	91–119	0.1483	100	90–118	101	91–117	0.3268
Glomerular filtration rate (mL/min/1.73 m^2^), SD	82.92	27.53	78.08	26.36	0.2250	76.08	25.43	76.70	25.40	0.3275
^*^Aspartate aminotransferase (IU/L), IQR	23	19–28	25	20–30	0.1571	24	20–30	25	20–30	0.1670
^*^Alanine aminotransferase (IU/L), IQR	20	15–28	23	17–33	0.1888	22	16–31	23	17–33	0.2071
^*^Gamma-glutamyl transpeptidase (IU/L), IQR	23	15–39	27	18–46	0.3226	26	17–45	27	18–45	0.5592
Smoking status *(N)*, %					< 0.0001					0.0002
Never	176,778	64.07%	22,310	69.38%		15,586	69.87%	15,562	69.76%	
Former	40,419	14.65%	4,914	15.28%		3,224	14.45%	3,472	15.56%	
Current	58,730	21.28%	4,931	15.34%		3,498	15.68%	3,274	14.68%	
Alcohol consumption *(N)*, %					< 0.0001					< 0.0001
Moderate	201,944	72.91%	25,646	79.52%		17,267	77.40%	17,763	79.63%	
Excessive	75,039	27.09%	6,603	20.48%		5,041	22.60%	4,545	20.37%	
Exercise *(N)*, %					< 0.0001					0.9552
Irregular	219,421	79.89%	24,754	77.20%		17,150	76.88%	17,155	76.90%	
Active	55,244	20.11%	7	22.80%		5,158	23.12%	5,153	23.10%	
Income *(N)*, %					< 0.0001					0.2044
Low	92,049	44.03%	10,991	45.70%		4,743	21.26%	4,671	20.94%	
Middle	45,456	22.22%	8,040	33.43%		7,305	32.75%	7,481	33.54%	
High	70,536	33.74%	5,018	20.87%		10,260	45.99%	10,156	45.53%	
Residence *(N)*, %					< 0.0001					0.3581
Urban area	95,013	45.37%	11,778	48.89%		10,725	48.08%	10,822	48.51%	
Rural area	114,391	54.63%	12,314	51.11%		11,583	51.92%	11,486	51.49%	
History of hypertension or medication *(N)*, %	76,466	27.61%	24,998	77.52%	< 0.0001	17,538	78.62%	17,011	76.26%	< 0.0001
History of diabetes or medication *(N)*, %	41,682	15.05%	15,901	49.31%	< 0.0001	10,134	45.43%	10,217	45.80%	0.4302
Charlson comorbidity index, SD	1.54	2.02	0.86	1.89	0.0118	1.06	1.82	0.93	1.96	0.0179
10-year ASCVD risk, %					< 0.0001					< 0.0001
Low	160,068	85.04%	5,646	17.56%		4,558	20.43%	4,025	18.04%	
Borderline	36,324	13.17%	5,222	16.25%		2,897	12.99%	3,826	17.15%	
Intermediate	69,584	25.23%	17,102	63.21%		11,280	50.56%	11,985	53.73%	
High	9,826	3.56%	4,169	12.97%		3,573	16.02%	2,472	11.08%	

* Triglyceride, High-density lipoprotein cholesterol, Fasting blood glucose, Aspartate aminotransferase, Alanine aminotransferase, and Gamma-glutamyl transpeptidase are expressed as the median value.

PS, Propensity score; SD, Standardized Difference; IQR, Interquartile range; SE, Standard error.

### Incidence of outcomes

A total of 2,624 participants experienced a MACE during the follow-up period (mean, 5.66 years; standard deviation, 0.93 years). The incidence of MACE was higher in statin non-users than that in users (10.94 vs. 9.82/1,000 person-years). Specifically, the incidence of ischemic stroke and CVD death was higher in statin non-users than that in users, whereas the incidence of MI was higher in statin users than that in non-users (Table 2).

**Table 2:** Incidence and hazard ratio of MACE.

**Among PS matched cohort**	**Statin non-users**		**Statin users**		**Hazard ratio**	**95% CI**	
**(*n* = 44,616)**	***n***	IR per 1,000 PY^*^	***n***	IR per 1,000 PY^*^			
MACE	1,406	10.94	1,218	9.82	0.90	0.84	0.98
MI	233	1.77	281	2.22	1.27	1.07	1.51
Ischemic stroke	1,165	9.02	952	7.63	0.85	0.78	0.93
CVD death	82	0.62	70	0.55	0.90	0.65	1.24

* Incidence rate per 1,000 person-year.

Hazard ratio (HR) was obtained with reference to the statin non-user group. CVD, cardiovascular disease; MACE, major adverse cardiovascular events; MI, myocardial infarction; CI, confidence interval.

A total of 4,490 participants experienced a MACE plus during the follow-up period. The incidence of MACE plus was higher in statin users than that in non-users (18.59 vs. 17.67/1,000 person-years). In particular, the incidence of TIA, unstable angina, and coronary revascularization was higher in statin users than in that non-users (Supplementary Table S2).

### Hazard ratios of MACE and MACE plus

The MACE HR was 0.90 (95% CI, 0.84–0.98) in statin users compared with that in non-users. Among MACEs, the risk of ischemic stroke was decreased, whereas the risk of MI was increased in statin users compared to that in non-users. There was no statistically significant effect of statins on cardiovascular death (Table 2).

Moreover, MACE plus HRs showed no significant difference between statin users and non-users. Among MACE plus, the risk of unstable angina was increased in statin users, whereas no significant effect of statin was observed on TIA and coronary revascularization (Supplementary Table S2).

### Hazard Ratio of MACE and MACE plus by 10-year ASCVD risk

According to the 10-year ASCVD risk categories, statin users exhibited the highest HR for MACE, compared to non-users, in the low-risk group. It was found that the higher the risk category, the lower the HR.

In the low-risk group, statin use significantly increased the risk of MACE (HR 1.80, 95% CI 1.29–2.52). Statin use especially increased the risk of MI (HR 4.14, 95% CI 1.98–8.67); however, the risk of ischemic stroke and cardiovascular death were not influenced by statin use (Table 3). Additionally, statin use increased the risk of MACE plus and its subcategories such as TIA, unstable angina, and coronary revascularization in the low-risk group (Supplementary Table S3).

**Table 3:** Hazard ratio of MACE by 10-year ASCVD risk categories.

**Among PS matched cohort**	**Low**			**Borderline**			**Intermediate**			**High**		
**(*n* = 44,616)**	**HR**	**95% CI**		**HR**	**95% CI**		**HR**	**95% CI**		**HR**	**95% CI**	
MACE	1.80	1.29	2.52	1.15	0.86	1.54	0.94	0.85	1.03	0.83	0.71	0.96
MI	4.14	1.98	8.67	1.08	0.63	1.86	1.29	1.02	1.62	1.10	0.77	1.56
Ischemic stroke	1.41	0.95	2.09	1.26	0.90	1.78	0.88	0.79	0.98	0.82	0.70	0.97
CVD death	1.59	0.36	7.10	1.17	0.20	7.03	1.09	0.71	1.68	0.82	0.47	1.40

Hazard ratio (HR) was obtained with reference to the statin non-user group. CVD, cardiovascular disease; ASCVD, atherosclerotic CVD; MACE, major adverse cardiovascular events; MI, myocardial infarction; CI, confidence interval.

In the borderline-risk group, the MACE HR of statin users were 1.15 compared to that of non-users, which was not significant; however, this was slightly less than the value determined in the low-risk group. Similar results were shown in MACE plus.

Most members of the study population belonged to the intermediate-risk group, for which the risk of MACE was decreased (HR 0.94, 95% CI 0.85–1.03); however, this difference was not statistically significant. Additionally, the risk of MI increased more, the risk of ischemic stroke was significantly reduced, and the incidence of cardiovascular death was not affected by statin use (Table 3). The risk of MACE plus was also decreased (HR 0.98, 95% CI 0.90–1.06); however, this change was not statistically significant. The risk of unstable angina showed a significant increase, while TIA and coronary revascularization did not differ significantly (Supplementary Table S3).

In the high-risk group, the risk of MACE was significantly reduced following statin use (HR 0.83, 95% CI 0.71–0.96). Although statin use increased the risk of MI, this difference was not statistically significant. Ischemic stroke incidence was significantly reduced by statin use (HR 0.82, 95% CI 0.70–0.97). The risk of MACE plus was decreased (HR 0.91, 95% CI 0.80–1.04); however, this difference was not statistically significant. In contrast, the risk of unstable angina showed a significant increase (HR 1.36, 95% CI 1.06–1.75).

### Subgroup analyses

We conducted subgroup analyses based on sex, age, and the incidence of hypertension or DM. In women, the risk of MACE was reduced by statin use; however, this risk was unchanged in men. The use of statins in individuals below 65 years of age and those with hypertension increased the risk of MACE. Additionally, the use of statins significantly reduced the risk of MACE in individuals with DM, while ischemic stroke risk was reduced by statin use, and MI risk was not affected by statin use in individuals with DM (Table 4). No differences were noted between men and women regarding MACE plus and the use of statins. All other results for MACE plus were similar to that of MACE (Supplementary Table S4).

**Table 4:** Hazard ratio of MACE by subgroups.

**Among PS matched cohort**	**MACE**				**MI**			**Ischemic stroke**			**CVD death**		
**(*n* = 44,616)**	***n***	**HR**	**95% CI**		**HR**	**95% CI**		**HR**	**95% CI**		**HR**	**95% CI**	
Male (*n =* 18,549)	1,241	0.95	0.85	1.06	1.53	1.23	1.90	0.84	0.73	0.95	0.90	0.59	1.36
Female (*n =* 26,067)	1,383	0.87	0.79	0.97	0.96	0.72	1.28	0.86	0.77	0.97	0.93	0.56	1.54
Age < 65 years (*n =* 29,774)	1,040	1.13	1.00	1.28	1.50	1.16	1.93	1.08	0.94	1.24	1.16	0.65	2.07
Age ≥65 years (*n =* 14,842)	1,584	0.87	0.78	0.96	1.18	0.92	1.51	0.82	0.74	0.92	0.92	0.62	1.35
No Diabetes mellitus (*n =* 24,265)	1,127	1.03	0.91	1.15	1.48	1.12	1.95	0.96	0.84	1.09	1.09	0.62	1.91
Diabetes mellitus (*n =* 20,351)	1,497	0.82	0.74	0.90	1.15	0.92	1.43	0.77	0.69	0.87	0.82	0.55	1.21
No Hypertension (*n =* 10,067)	299	1.44	1.14	1.82	1.82	1.07	3.08	1.39	1.07	1.80	1.35	0.43	4.25
Hypertension (*n =* 34,549)	2,325	0.87	0.80	0.94	1.23	1.02	1.48	0.81	0.74	0.89	0.89	0.64	1.24

Hazard ratio (HR) was obtained with reference to the statin non-user group. CVD, cardiovascular disease; MACE, major adverse cardiovascular events, MI, myocardial infarction; CI, confidence interval.

We also investigated the HR of MACE based on the LDL-C levels and found that the higher the LDL-C levels, the lower the risk of MACE associated with statin use. Meanwhile, the risk of MACE was significantly reduced with statins when LDL-C >160 mg/dL. The risk of ischemic stroke was significantly reduced with statins when LDL-C levels were >100 mg/dL. However, the incidence of MI was not affected by statin use at any LDL-C level (Table 5). The MACE plus results were similar to those of MACE (Supplementary Table S5). Finally, we found that new-onset DM incidence was higher in statin users (90.18 vs. 59.73/1,000 person-years), the HR for which was 1.59 (95% CI, 1.53–1.66) (Table 6).

**Table 5:** Hazard ratio of MACE by LDL categories.

**Among PS matched cohort**	**MACE**				**MI**			**Ischemic stroke**			**CVD death**		
**(*n* = 44,616)**	***n***	**HR**	**95% CI**		**HR**	**95% CI**		**HR**	**95% CI**		**HR**	**95% CI**	
LDL < 70 mg/dL	315	1.10	0.87	1.39	1.42	0.88	2.30	1.10	0.84	1.44	0.67	0.30	1.48
(*n =* 4,616)													
70 ≤ LDL < 100 mg/dL	656	1.07	0.92	1.25	1.83	1.28	2.63	0.97	0.82	1.15	1.09	0.58	2.05
(*n =* 10,861)													
100 ≤ LDL < 130 mg/dL	815	0.80	0.69	0.92	1.07	0.77	1.49	0.77	0.65	0.90	0.61	0.30	1.22
(*n =* 13,377)													
130 ≤ LDL < 160 mg/dL	564	0.84	0.71	1.00	0.98	0.65	1.48	0.82	0.68	0.99	1.36	0.66	2.78
(*n =* 10,401)													
160 ≤ LDL < 190 mg/dL	271	0.74	0.58	0.93	0.78	0.46	1.32	0.72	0.55	0.94	0.55	0.22	1.36
(*n =* 5,361)													

Hazard ratio (HR) was obtained with reference to the statin non-user group. CVD, cardiovascular disease; MACE, major adverse cardiovascular events, MI: myocardial infarction; CI, confidence interval; PS, propensity score.

**Table 6:** Hazard ratio of new-onset DM by statin use.

**Among no diabetes mellitus**	**New-onset diabetes mellitus** ^*^					
**in the PS matched cohort (*n* = 24,265)**	***n***	**Person-year**	IR per 1,000 PY^**^	**HR**	**95% CI**	
Statin non-user (*n* = 12,174)	3,738	63,330.57	59.73	1		
Statin user (*n* = 12,091)	5,135	56,940.75	90.18	1.59	1.53	1.66

* New-onset diabetes mellitus was defined as when the subjects had a diagnostic code for diabetes mellitus (DM) (ICD-10 E10 ~E14), had a fasting blood glucose level of 126 mg/dL or higher, or had been prescribed hypoglycemic drug (ATC code A10B) or insulin (ATC code A10A).

** Incidence rate per 1,000 person-year; PS, propensity score; CI, confidence interval.

## Discussion

In this retrospective cohort study, we demonstrated that statin therapy was associated with reduced MACE among Koreans for the primary prevention of CVD. However, this preventive effect appeared only in the high 10-year ASCVD-risk group, whereas statin therapy increased the risk of MACE in Koreans in the low-risk group. Through a subgroup analysis, we found that the benefits of statins use increased when the individuals were at risk for CVD due to factors such as old age (≥65 years), hypertension, DM, and higher LDL-C levels (160–189 mg/dL). Moreover, statin use increased the incidence of new-onset DM. These results suggest that statin therapy should be customized to patients based on their individual and race-specific 10-year ASCVD risk factors.

Previously, the Cholesterol Treatment Trialists' Collaboration reported that the reduction in LDL-C levels with statin therapy reduced the risk of major vascular events in individuals with no previous history of vascular disease, and even in those with a 5-year risk < 10% (24). Similarly, a meta-analysis by the US Preventive Services Task Force on the use of statins as the primary preventive method using 13 randomized clinical trials indicated that statin use in adults with no history of CVD significantly reduced the incidence of composite CVD outcomes (25). Our study agrees with these previous reports, showing that statin use for primary prevention reduced the HRs of MACE.

However, upon applying the Korean-specific 10-year ASCVD risk estimation, statin use did not confer protection against CVD in the low- to intermediate-risk populations, unlike those reported in previous randomized controlled trials (4, 5, 7, 24). We postulated that these results may be due to background population risk. The 2019 ACC/AHA Guidelines on the Primary Prevention of CVD have used the US-derived pooled cohort equation to estimate 10-year ASCVD risk. However, the incidence and risk factor levels of coronary artery disease in the Korean population have been reported to be lower than those in the United States (26, 27). Therefore, simply employing the ACC/AHA 10-year ASCVD score would theoretically overestimate the cardiovascular risk in Koreans (17). In addition, the recalibration coefficients of the Framingham coronary heart disease risk score published by the International Atherosclerosis Society, which considers background population risk, revealed that the coefficients in China are much lower than those in urban India. Therefore, the number needed to treat (NNT) to prevent one ASCVD in people with a similar risk factor profile would be much higher in China, which is similar to the findings in Korea, than in urban India (19, 21). Meanwhile, a nationwide population-based study revealed that in 2015, although the ischemic mortality rate was relatively low, the prevalence and cumulative incidence of ischemic stroke (18.62 and 6.28 per 1,000 individuals, respectively) were higher than those of MI (5.61 and 2.36 per 1,000 individuals, respectively) in Korea (28). Thus, the NNT to prevent MI in individuals using statins might be higher than that needed to prevent ischemic strokes. Accordingly, we employed the Korean-specific 10-year ASCVD risk estimate to evaluate the population-specific risk and found that the net beneficial effect of statins with respect to protection from CVD in Koreans may differ from that in Americans (29).

In our study, the risk of MACEs resulting from the use of statins increased as the risk of ASCVD decreased. MACE risk is higher among low-risk patients (those without hypertension or DM and younger than 65 years) who initiated statins than among those who did not. Similarly, a previous study reported no association between statins and a reduction in CVD events in individuals without type 2 DM (in contrast to those with type 2 DM) (30). Additionally, a Korean population-based study using their prediction model (risk score 0–13, C-index = 0.716), which took into account age, sex, hypertension, DM, anemia, C-reactive protein, and the extent of non-obstructive coronary artery disease, showed that statin use was associated with a lower risk of cardiovascular events in the high- or very high-risk groups (risk score ≥7), but, not in the low- and intermediate-risk groups (risk score < 7) (31). Further, our subgroup analysis revealed that the HRs of MACE were significantly reduced only when the LDL-C levels were above 160 mg/dL. Therefore, we postulated that the anti-atherosclerotic functions and pleiotropic effects of statins may be beneficial only in populations with a high CVD risk or elevated LDL-C levels. Taken together, these findings suggest that the use of statins for primary prevention in low-to intermediate-risk individuals may not be effective in the Korean population.

It should be noted that the detrimental effects of statins in the low-ASCVD risk group may also be related to the occurrence of DM, as it is a well-known risk factor for CVD (32). Moreover, several studies have shown that statin therapy increases the incidence of DM (33–35), with the underlying mechanisms involving the following: calcium channels of pancreatic β cells that are related to insulin secretion; reduced potential of glucose transporter 4, which can lead to hyperglycemia and hyperinsulinemia; or altered intracellular signaling pathways owing to the reduction in major downstream signaling molecules, such as coenzyme Q10 (36). In addition, genetic variations in the statin-binding site (lipid-lowering alleles in 3-hydroxy-3-methylglutaryl coenzyme A reductase) are linked to increased BMI and an increased risk of type 2 DM (37). Furthermore, statin use resulted in the DNA methylation of the cg6500161 alleles of ABCG1, a member of the ATP-binding cassette (ABC) protein family that is responsible for removing surplus cholesterol from the peripheral tissues and transporting it to the liver. This DNA methylation was positively associated with increased fasting blood glucose in non-diabetic subjects (38). Our study showed a higher incidence of new-onset DM in statin users compared to non-users, and a retrospective cohort study by the Korean National Health Insurance System (NHIS) showed that a change from normal fasting glucose to diabetic glucose leads to increased MI, stroke, and all-cause mortality in adults over 40 years of age without diabetes or CVD (39). Therefore, statin use may increase fasting blood glucose levels or induce diabetes development in the low ASCVD-risk groups. Hence, if the ASCVD risk is not relatively high, the beneficial effect of statins does not appear to outweigh the risk of developing DM, which could lead to MACE.

Several limitations were noted in this study. First, we could not assess the coronary atherosclerosis burden that may be associated with the development of CVD. A recent study showed that atherosclerotic burden is not correlated with the spectrum of LDL-C levels (40). In a stratified analysis according to LDL-C levels in 23,143 patients with no history of cardiovascular disease but with chest-related symptoms, any coronary plaques (calcified or non-calcified) were detected at baseline regardless of statin use or LDL level. Coronary plaques were also found in the LDL-C group of < 77 mg/dL, and when plaques were identified, the incidence of cardiovascular events was higher when the LDL-C level was < 77 mg/dL compared to the group with high LDL-C level (40). That is, some populations may have had coronary atherosclerotic changes, even if they were classified as low-risk due to low LDL-C levels and no history of ASCVD. Our study showed relatively high incidence of MI on low-risk group, it can be expected that a higher CVD event rate may appear if DM incidence was elevated by statin use in a group with coronary plaques already present although classified as a low-risk group. Second, we only used data available from public sources, which may have affected our conclusions, i.e., diagnoses reported in insurance claims may differ slightly from the actual disease or condition of the patient. However, previous studies comparing positive predictive values between ICD-10 code-based, diagnostic claims, and medical records, reported numerical positive and predictive values for DM (72.3 to 87.2%) (41), acute MI (over 70%) (42), ischemic stroke (83.4%) (43), and overall diagnoses (70%) (44) that were similar to those observed in our study. Third, confounding by indication may have occurred (45). More statins would have been prescribed for patients with a family history of cardiovascular disease or chest related symptoms at the time of prescription. Or individuals in the group treated with statins may have been more susceptible to symptoms and were likely tested more frequently. If a patient was suspected of having a myocardial infarction due to chest pain and underwent percutaneous coronary angioplasty, it might be included as MACE or MACE plus according to the definition of this study, even if there was no significant atherosclerotic lesion, resulting in a high HR. However, to conduct research only among individuals who were not at a high-risk of CVD at the time of prescription, we excluded those individuals who had a history of CVD 2years prior to the index date. Furthermore, we performed a PS matching to reduce group differences. Nevertheless, a randomized clinical trial with a larger sample size and longer follow-up is needed to achieve a more reliable conclusion, particularly for low- to moderate-risk populations (46). Finally, the follow-up period for this study was not sufficiently long (mean duration of 5.66 years) to assess MACEs, considering that the study population had a low CVD risk; therefore, a substantially longer follow-up period is required. The relatively short follow-up period was partly owing to the availability of LDL-C data, which were first incorporated into the National Health Examinations in 2009.

To the best of our knowledge, this is the first study to investigate the role of statins for primary prevention in low CVD-risk Korean populations using recalibrated, pooled, cohort equations. Considering the necessity of evaluating statin based on diverse races, our study provides insight into the importance of population-specific statin therapy for the primary prevention of CVD. In addition, our study included half a million Korean individuals who participated in a National Health Examination; therefore, the results are likely representative of individuals in low-risk Korean populations despite any potential inaccuracies.

## Conclusions

Our novel findings indicate that statin use is not associated with MACE reduction in low- to intermediate-risk Koreans within groups with low ischemic mortality rate. Although this study had several limitations, the results suggest that individuals with LDL-C levels < 190 mg/dL and those with a low risk of ASCVD should consider statin therapy only when the risk of CVD is obvious as determined using an appropriate ASCVD risk tool. More conclusive evidence is needed on the use of statin therapy for the primary prevention of CVD, particularly in low-risk individuals.

## Data availability statement

The original contributions presented in the study are included in the article/Supplementary materials, further inquiries can be directed to the corresponding author.

## Ethics statement

The studies involving human participants were reviewed and approved by the Institutional Review Board of Seoul National University Bundang Hospital (IRB of SNUBH) approved the current study (approval number: X-1611-372-905). Written informed consent for participation was not required for this study in accordance with the National Legislation and the Institutional Requirements.

## Author contributions

IR: conceptualization, methodology, data curation, writing–original draft, and writing–review and editing. JK: conceptualization, software, formal analysis, writing–review and editing, supervision, and funding acquisition. HP: conceptualization and methodology. SO: formal analysis. SK: data curation and investigation. HK: formal analysis and writing–review and editing. All authors contributed to the article and approved the submitted version.

## Funding

This study was supported by Grant No. 12-2013-005 from the Seoul National University Bundang Hospital (SNUHBH) Research Fund. The funder of the study had no role in the design and conduct of the study, collection, management, analysis and interpretation of the data, preparation, review or approval of the manuscript, decision to submit the manuscript for publication.

## Conflict of interest

The authors declare that the research was conducted in the absence of any commercial or financial relationships that could be construed as a potential conflict of interest.

## Publisher's note

All claims expressed in this article are solely those of the authors and do not necessarily represent those of their affiliated organizations, or those of the publisher, the editors and the reviewers. Any product that may be evaluated in this article, or claim that may be made by its manufacturer, is not guaranteed or endorsed by the publisher.
