# Supplementary material for: Do statins benefit low-risk population for primary prevention of atherosclerotic cardiovascular disease: A retrospective cohort study
Source: Front Med (Lausanne). 2022 Nov 3;9:1024780. doi: 10.3389/fmed.2022.1024780 (PMC9669657; doi:10.3389/fmed.2022.1024780)
Supplement: Supplementary file 1 [file Data_Sheet_1.pdf]

Do Statins Benefit Low-risk Population for Primary Prevention of Atherosclerotic Cardiovascular Disease? : A Retrospective Cohort Study

In sun Ryou[1], Ju Young Kim[2*], Hwa Yeon Park[2], Sohee Oh[3], Sehun Kim[4], Hwa Jung Kim[5]

[1]Department of Family Medicine, Ewha Womans University Medical Center, Ewha Womans University School of Medicine, Seoul 07804, Republic of Korea.

[2]Department of Family Medicine, Seoul National University Bundang Hospital and Seoul National University College of Medicine, Gyeounggi-do, 13620, Republic of Korea.

[3]Department of Biostatistics, Seoul Metropolitan Government Seoul National University Boramae Medical Center, Seoul 07061, Republic of Korea.

[4]Cardiovascular Center, Hallym University Medical Center, Seoul 07247, Republic of Korea.
[5]Department of Preventive Medicine, Ulsan University College of Medicine, Department of Clinical Epidemiology and Biostatistics, ASAN Medical Center, Seoul 05505, Republic of Korea

*** Correspondence:**
Ju Young Kim
kabbey1@snu.ac.kr

Supplementary Table 1. Definitions of MACE and MACE plus

Definition	ICD-10 codes	Other codes	ATC codes
**Myocardial infarction**			
Acute myocardial infarction	I21.x (0.1.2.3.4.9)		
Subsequent myocardial infarction	I22.x (0,1,8,9)		
Complications of following myocardial infarction	I23.x (0~6,8)		
Other forms of acute ischemic heart disease	I24.8		
Acute ischemic heart disease, unspecified	I24.9		
**Stroke**			
Cerebral infarction	I63.x (0~9)		
Stroke, not hemorrhage nor infarction	I64.		
**Transient ischemic attack and related syndromes**	G45.x (0,1,2,3,4,8,9)		
**Unstable angina**	I20.0		
**Coronary revascularization procedures**			
Aortocoronary venous bypass graft angiography		HA370, HA680~HA682	
Percutaneous transluminal coronary angiography		M6551, M6552	
Percutaneous transcatheter placement of intracoronary		M6561~ M6564	

Percutaneous transluminal coronary atherectomy	M6571, M6572
Percutaneous intravascular installation of metallic stent<br>Percutaneous intravascular installation of metallic stent atherectomy	M6620
Percutaneous thrombus removal-mechanical thrombolysis	M6633
Percutaneous thrombus removal-thrombolytic treatment-coronary artery	M6634
Vascular bypass operation (aorta-coronary)	O1641, O1642, O1647
Coronary endarterectomy	O1830
Thrombectomy (artery), chest	O2053
Thrombectomy (deep vein), chest	O2057
Angioplasty (end-to-end anastomosis)-by thoracotomy	OA631, OB631
Angioplasty (with patch graft)-by thoracotomy	OA634, OA635, OB634, OB635
**Insulin or oral hypoglycemic agents**	
Insulin	A10A
Oral hypoglycemic agents	A10B
**Statin prescription**	
Atorvastatin	C10AA05
Rosuvastatin	C10AA07

Simvastatin	C10AA01
Lovastatin	C10AA02
Fluvastatin	C10AA04
Pitavastatin	C10AA08
Pravastatin	C10AA03

MACE: major adverse cardiac event

Supplementary Table 2. Incidence and hazard ratio of MACE plus

Among PS-matched cohort (n=44,616)	Statin non-users			Statin users			Hazard ratio	95% CI	
	n	PY	IR per 1,000 PY*	n	PY	IR per 1,000 PY*			
MACE plus	2,257	125,581.00	17.97	2,233	120,141.39	18.59	0.97	0.91	1.03
TIA	492	130,900.68	3.76	530	125,843.55	4.21	1.12	0.99	1.27
Unstable angina	477	130,973.68	3.64	666	125,473.13	5.31	1.46	1.30	1.64
Coronary Revascularization	453	131,185.77	3.45	444	126,302.48	3.52	1.03	0.90	1.18

Hazard ratio was calculated with reference to the statin non-user group.

PY: person-years; PS: propensity score; MACE: major adverse cardiac event; TIA: transient ischemic attack; CI: confidence interval

Supplementary Table 3. HR for MACE plus by 10-year Atherosclerotic cardiovascular disease risk categories

Among PS-matched cohort	Low			Borderline			Intermediate			High		
(n=44,616)	HR	95% CI		HR	95% CI		HR	95% CI		HR	95% CI	
MACE plus	1.74	1.36	2.22	1.15	0.92	1.43	0.98	0.90	1.06	0.91	0.80	1.04
TIA	1.51	1.02	2.25	1.12	0.77	1.63	1.11	0.95	1.31	1.11	0.84	1.46
Unstable angina	2.26	1.54	3.33	1.43	0.98	2.10	1.49	1.27	1.74	1.36	1.06	1.75
Coronary revascularization	2.55	1.46	4.46	0.78	0.50	1.22	1.03	0.87	1.23	1.08	0.83	1.40

HR was calculated with reference to the statin non-user group.

HR: hazard ratio; PS: propensity score; TIA: transient ischemic attack; MACE: major adverse cardiac event; CI: confidence interval

Supplementary Table 4. HR for MACE by subgroup

Among PS-matched cohort	MACE				MI			Stroke			CVD death		
(n=44,616)	n	HR	95% CI		HR	95% CI		HR	95% CI		HR	95% CI	
Male (n=18,549)	1,241	0.95	0.85	1.06	1.53	1.23	1.90	0.84	0.73	0.95	0.90	0.59	1.36
Female (n=26,067)	1,383	0.87	0.79	0.97	0.96	0.72	1.28	0.86	0.77	0.97	0.93	0.56	1.54
Age < 65 years (n=29,774)	1,040	1.13	1.00	1.28	1.50	1.16	1.93	1.08	0.94	1.24	1.16	0.65	2.07
Age ≥ 65 years (n=14,842)	1,584	0.87	0.78	0.96	1.18	0.92	1.51	0.82	0.74	0.92	0.92	0.62	1.35
Without diabetes mellitus (n=24,265)	1,127	1.03	0.91	1.15	1.48	1.12	1.95	0.96	0.84	1.09	1.09	0.62	1.91
Diabetes mellitus (n=20,351)	1,497	0.82	0.74	0.90	1.15	0.92	1.43	0.77	0.69	0.87	0.82	0.55	1.21

Without hypertension (n=10,067)	299	1.44	1.14	1.82	1.82	1.07	3.08	1.39	1.07	1.80	1.35	0.43	4.25
Hypertension (n=34,549)	2,325	0.87	0.80	0.94	1.23	1.02	1.48	0.81	0.74	0.89	0.89	0.64	1.24

HR was calculated with reference to the statin non-user group.

HR: hazard ratio; PS: propensity score; MI: myocardial infarction; MACE: major adverse cardiac event; CI: confidence interval; CVD: cardiovascular disease

Supplementary Table 5. HR for MACE plus by subgroup

Among PS-matched cohort (n=44,616)	MACE plus				TIA			Unstable angina			Coronary revascularization		
	n	HR	95% CI		HR	95% CI		HR	95% CI		HR	95% CI	
Male (n=18,549)	1,831	1.03	0.94	1.13	1.14	0.93	1.39	1.71	1.45	2.01	1.23	1.04	1.46
Female (n=26,067)	2,105	0.93	0.85	1.01	1.11	0.95	1.30	1.26	1.06	1.49	0.82	0.67	1.01
Age < 65 years (n=29,774)	1,720	1.13	1.03	1.25	1.13	0.95	1.35	1.52	1.29	1.79	1.07	0.89	1.30
Age ≥ 65 years (n=14,842)	2,216	0.95	0.88	1.04	1.21	1.02	1.44	1.52	1.28	1.80	1.08	0.90	1.30
Without diabetes mellitus (n=24,265)	1,740	1.08	0.98	1.18	1.13	0.95	1.34	1.90	1.58	2.28	1.22	0.99	1.51
Diabetes mellitus	2,196	0.89	0.82	0.97	1.12	0.94	1.33	1.20	1.03	1.40	0.92	0.78	1.09

(n=20,351)													
Without hypertension (n=10,067)	502	1.49	1.25	1.78	1.61	1.18	2.20	1.56	1.11	2.20	1.24	0.83	1.86
Hypertension (n=34,549)	3,434	0.93	0.87	0.99	1.06	0.92	1.21	1.47	1.30	1.67	1.03	0.89	1.18

HR was calculated with reference to the statin non-user group.

HR: hazard ratio; PS: propensity score; MACE: major adverse cardiac event; CI: confidence interval; TIA: transient ischemic attack

Supplementary Table 6. HR for MACEs by LDL-C category

Among PS-matched cohort (n=44,616)	**MACE**				**MI**			**Stroke**			**CVD death**		
	n	HR	95% CI		HR	95% CI		HR	95% CI		HR	95% CI	
LDL-C < 70 mg/dL (n=4,616)	315	1.10	0.87	1.39	1.42	0.88	2.30	1.10	0.84	1.44	0.67	0.30	1.48
70 ≤ LDL-C < 100 mg/dL (n=10,861)	656	1.07	0.92	1.25	1.83	1.28	2.63	0.97	0.82	1.15	1.09	0.58	2.05
100 ≤ LDL-C < 130 mg/dL (n=13,377)	815	0.80	0.69	0.92	1.07	0.77	1.49	0.77	0.65	0.90	0.61	0.30	1.22
130 ≤ LDL-C < 160 mg/dL (n=10,401)	564	0.84	0.71	1.00	0.98	0.65	1.48	0.82	0.68	0.99	1.36	0.66	2.78
160 ≤ LDL-C < 190 mg/dL (n=5,361)	271	0.74	0.58	0.93	0.78	0.46	1.32	0.72	0.55	0.94	0.55	0.22	1.36

HR was calculated with reference to the statin non-user group.

HR: hazard ratio; LDL-C: low-density lipoprotein cholesterol; PS: propensity score; MI: myocardial infarction; MACE: major adverse cardiac event; CI: confidence interval; CVD: cardiovascular disease

Supplementary Table 7. HR for MACE plus by LDL-C category

Among PS-matched cohort	MACE plus				TIA			Unstable angina			Coronary revascularization		
(n=44,616)	n	HR	95% CI		HR	95% CI		HR	95% CI		HR	95% CI	
LDL-C < 70 mg/dL (n=4,616)	445	1.14	0.94	1.39	0.87	0.59	1.28	1.91	1.33	2.75	1.21	0.80	1.81
70 ≤ LDL-C < 100 mg/dL (n=10,861)	1,011	1.18	1.04	1.33	1.37	1.07	1.76	1.71	1.36	2.16	1.59	1.23	2.07
100 ≤ LDL-C < 130 mg/dL (n=13,377)	1,189	0.90	0.80	1.01	1.13	0.89	1.43	1.32	1.06	1.65	0.94	0.73	1.21
130 ≤ LDL-C < 160 mg/dL (n=10,401)	861	0.86	0.75	0.99	0.96	0.75	1.24	1.28	0.99	1.66	0.81	0.60	1.09
160 ≤ LDL-C < 190 mg/dL (n=5,361)	430	0.76	0.63	0.92	1.29	0.86	1.93	0.88	0.61	1.28	0.49	0.34	0.73

HR was calculated with reference to the statin non-user group.

HR: hazard ratio; LDL-C: low-density lipoprotein cholesterol; PS: propensity score; TIA: transient ischemic attack; MACE: major adverse cardiac event;

CI: confidence interval

Supplement A. The Korean Risk Prediction Model

K.J. Jung et al., Atherosclerosis 242 (2015) 367–75.

The recalibrated equation for the Korean population was calculated by adopting the “pooled cohort estimation (PCE) for accessing ASCVD risk” developed by ACC/AHA in 2013.

As per the method proposed by D'Agostino et al. (2001), the ACC/AHA 2013 PCE was recalibrated for the Korean Heart Study (KHS) cohort, which included 200,010 Korean adults aged 40–79 years and who did not have a diagnosis of ASCVD.

$$f(x,M) = \beta_1(x_1 - M_1)+...+ \beta_p(x_p - M_p)$$

$$P = 1-S(t)^{\exp(f[x,M])},$$

where *S(t)* is the baseline survival rate at 10 years, *β1...βp* are the regression coefficients*, x1...xp* represent an individual’s risk factors, and *M1...Mp* represent the mean values of the risk factors in the studied population.

In the recalibrated ACC/AHA equations, the coefficients were taken from the ACC/AHA equations' Cox model, whereas the risk factors for the ACC/AHA equations were replaced by the mean values of the risk factors from the KHS cohort. The ACC/AHA average incidence rate, S0(t), was replaced by the average incidence rate of the KHS cohort. Risk scores for this ASCVD risk equation are as follows.

For men, the risk score (KRS-M) was defined using the following steps:

KMSUM = 9.362 × AGE + 2.425 × AGESQ + 6.409 × TC - 1.430 × AGETC -

3.843 × HDL + 0.810 × AGEHDL + 18.589 × TRSBP - 4.116 × AGETRSBP +

18.541 × UNSBP - 4.112 × AGEUNSBP + 2.464 × CUSMOK - 0.503 × LAGESMOK

$+ 0.410 \times DM.$

All variables except for smoking status and diabetes were used in log format.

AGESQ is the squared age, *TC* is total cholesterol, *HDL* is HDL-cholesterol, *TRSBP* is treated systolic blood pressure, *UNSBP* is untreated systolic blood pressure, *CUSMOK* is current smoker, and *DM* is diabetes mellitus.

When an age interaction is present with lipids or BP, the natural log of age is multiplied by the natural log of the lipid or BP, and the result is multiplied by the parameter estimate. AGETC represents the interaction of AGE and TC.

$$\textbf{MSUM} = 9.362 \times 3.902 + 2.425 \times 15.253 + 6.409 \times 5.263 - 1.430 \times 20.538 - 3.843 \times 3.847 + 0.810 \times 15.014 + 18.589 \times 0.262 - 4.116 \times 1.049 + 18.541 \times 4.555 - 4.112 \times 17.754 + 2.464 \times 0.496 - 0.503 \times 1.923 + 0.410 \times 0.101$$

For example, according to Table 2, the mean age of men in the KHS was 50.13 years and the logarithmic value was 3.902, so AGE was substituted by 3.902 in the MSUM equation.

$$x = KMSUM - MSUM$$

$$y = \exp(x)$$

Finally, the absolute 10-year risk of ASCVD is KHS_M = $(1 - 0.96427^{y})$, where 0.96427 is the baseline survival rate for men.

For women, the risk score (KRS-W) was defined similarly.

KWSUM = -9.519 × AGE + 3.417 × AGESQ + 0.320 × TC - 0.476 × HDL +

13.402 × TRSBP - 2.889 × AGETRSBP + 13.291 × UNSBP - 2.876 × AGEUNSBP

+ 0.415 × CUSMOK + 0.424 × DM.

WSUM = -9.519 × 3.935 + 3.417 × 15.507 + 0.320 × 5.284 - 0.476 × 3.965 +

13.402 × 0.329 - 2.889 × 1.326 + 13.291 × 4.483 - 2.876 × 17.616 + 0.415 × 0.045

+ 0.424 × 0.072

x = KWSUM – WSUM

y = exp(x).

Finally, the absolute 10-year risk of ASCVD is KHS_W = (1 - 0.96963^{y}), where 0.96963 is the baseline survival rate for women.

[Table 1]

Equation parameters of the Korean Risk Prediction Model in men and women aged 40–79 years from the Korean heart study (1997–2012).

	Men			Women		
	KHS	ACC/AHA (white)	ACC/AHA (AA)	KHS	ACC/AHA (white)	ACC/AHA (AA)
	Coefficient (SE)			Coefficient (SE)		
Ln age	9.36 (4.41)	10.21 (2.33)	3.97 (0.08)	−9.52 (4.73)	−16.12 (4.91)	11.00 (3.65)
Ln age square	2.42 (0.42)	–	–	3.42 (0.58)	2.52 (0.56)	–
Ln total cholesterol	6.41 (1.60)	7.92 (1.64)	0.69 (0.07)	0.32 (0.08)	1.81 (2.24)	0.27 (0.08)
Ln age * Ln total cholesterol	−1.43 (0.40)	−1.81 (0.41)		–	−0.37 (0.55)	–
Ln HDL-cholesterol	−3.84 (1.26)	−4.35 (1.28)	−0.59 (0.05)	−0.48 (0.06)	−2.32 (1.69)	−1.27 (1.73)
Ln age * Ln HDL-cholesterol	0.81 (0.31)	0.94 (0.32)	–	–	0.46 (0.42)	0.20 (0.43)
Ln treated SBP	18.59 (2.00)	2.04 (0.08)	2.04 (0.08)	13.40 (2.58)	1.70 (0.10)	8.08 (2.55)
Ln age * Ln treated SBP	−4.12 (0.50)	–	–	−2.89 (0.64)	–	−1.58 (0.63)
Ln untreated SBP	18.54 (2.00)	2.01 (0.08)	2.01 (0.08)	13.29 (2.63)	1.64 (0.10)	8.18 (2.61)
Ln age * Ln untreated SBP	−4.11 (0.50)	–	–	−2.88 (0.65)	–	−1.62 (0.64)
Current smoker	2.46 (0.60)	2.52 (0.59)	0.45 (0.02)	0.42 (0.06)	1.45 (1.45)	0.44 (0.06)
Ln age * current smoker	−0.50 (0.15)	−0.52 (0.15)	–	–	−0.25 (0.36)	–
Diabetes[a]	0.41 (0.03)	0.41 (0.03)	0.41 (0.03)	0.42 (0.04)	0.43 (0.04)	0.43 (0.04)
Baseline survival	0.96427	0.96394	0.96346	0.96963	0.96928	0.97001
AUC	0.734(0.727–0.740)	0.732(0.725–0.739)	0.731(0.725–0.738)	0.741(0.733–0.750)	0.741(0.733–0.749)	0.741(0.733–0.749)

Abbreviations: KHS, Korean Heart Study; Ln, natural log; HDL, High-density lipoprotein; LDL, Low-density lipoprotein; SBP, systolic blood pressure; SE, standard error; AUC, Area Under the receiver operation characteristic Curve.

[a] Diabetes was defined as fasting serum glucose >126 mg/dL or diabetic treatment history.

[Table 2]

Baseline characteristics of study participants aged 40–79 years from the Korean heart study (1996–2001), N = 200,010.

	Men (N = 119,715)	Women (N = 80,295)
	Mean (SD)	Mean (SD)
Age, year	50.13 (7.94)	51.81 (8.12)
Body Mass Index, kg/m^2	23.84 (2.69)	23.72 (3.03)
Total cholesterol, mg/dL	196.84 (34.79)	201.22 (37.44)
HDL-cholesterol, mg/dL	47.98 (10.88)	54.23 (12.83)
LDL-cholesterol, mg/dL	120.14 (32.54)	123.59 (34.04)
Systolic blood pressure, mmHg	124.79 (17.61)	124.49 (20.30)
Insurance premium, Korean won[a]	145,449 (158,873)	131,308 (142,791)
	%	%
Statin use	4.25	2.88
Smoking status		
Ex smokers	26.46	4.10
Current smokers	49.40	4.49
Diabetes[b] (yes)	10.49	7.43
Regular exercise (yes)	53.15	39.35

Abbreviations: HDL, high-density lipoprotein; LDL, low-density lipoprotein; SD, standard deviation.

[a] A measure of socioeconomic status was the health insurance premium per year, in South Korean 'Won' (1,129 Won = 1.00 US Dollar).

[b] Diabetes was defined as fasting serum glucose ≥126 mg/dL or a history of treatment for diabetes.

Reference: K.J. Jung et al., The ACC/AHA 2013 pooled cohort equations compared to a Korean Risk Prediction Model for atherosclerotic cardiovascular disease, Atherosclerosis 242 (2015) 367–375.

Supplement B. Sample data and program produced by SAS

```
/*****************************************************************************
**/
/** Create random dates for demonstration
**/
/** Exposed individuals will have an eligibility date (elig_date) and a treatment
exposure date (exposure_date) **/
/** Unexposed individuals will only have an eligibility date (elig_date)
**/
/*****************************************************************************
**/
DATA exposed(keep=grp elig_date exposure_date) unexposed(keep=grp
elig_date);
study_st='01Jan2010'd; /*observation (study) start date*/
study_end='31Dec2011'd;/*observation (study) end date*/
offset = (study_end-study_st+1)/4;/*offset only used for date
generating */
do i = 1 to 500;
grp=1;
Elig_date=study_st + int(ranuni(21)*offset);
Exposure_date=Elig_date + int(ranuni(321)*offset);
output exposed;
end;
do i = 1 to 2500;
grp=0;
Elig_date=study_st + int(ranuni(21)*offset);
output Unexposed;
end;
format study_st study_end Elig_date Exposure_date date9.;
RUN;
/** Step 1: Calculate time between eligibility and treatment exposure
```

```
(TimeToContc) for exposed **/
DATA Exposed_;
set exposed;
TimeToContc=intck('month',elig_date,exposure_date);
RUN;
/** Step 2: Output oneway Frequency table for event date and TimeToContc
for exposed **/
PROC FREQ DATA=exposed_;
table timetocontc;
ods output onewayFREQs=exp_time;
RUN;
PROC FREQ DATA=exposed_;
format exposure_date MONYY.;
table exposure_date;
ods output onewayFREQs=exposed_edate;
RUN;
/* Step 3: Create global macro variable representing number of
observations in unexposed DATA set **/
PROC SQL noprint;
select count(*)
into: num
from unexposed;
quit;
%put &num; /* check number of unexposed */
/* Step 4: Determine number of unexposed individuals per bins (cutpoints)
based on the distribution of the exposed
using the number of unexposed as the denominator
**/
DATA exp_time_;
set exp_time;
```

```
cutpt=round((CumPercent/100)*&num);/* this creates the number of
unexposed within each FREQuency bin based
on the
number of exposed in the ds */
keep cutpt;
RUN;
/** Step 5: SORT, TRANPOSE, and merge number of unexposed needed per bin
(cutpoints) to bins created from the
exposed Frequency distribution
**/
PROC SORT DATA=exp_time_;
by cutpt;
RUN;
PROC TRANSPOSE DATA=exp_time_ out=trans_exptime(drop=_name_) prefix=cutpt;
var cutpt ;
RUN;
PROC TRANSPOSE DATA=Exp_time out=trans_Expcontact(drop= _name_)
prefix=c_timetcntc;
var timetocontc;
RUN;
DATA Exp_pct;
set trans_exptime;
dummy=1;
RUN;
DATA Exp_contact;
set trans_Expcontact;
dummy=1;
RUN;
DATA trans_merge_exp;
merge exp_pct exp_contact;
```

```
by dummy;
RUN;
PROC SORT;
by dummy;
RUN;
/** Step 6: Randomly merge bins and number of unexposed needed per bin to
the unexposed Dataset **/
DATA unexposed_premerge;
set unexposed;
dummy=1;
a = UNIFORM(-32);
RUN;
PROC SORT DATA=unexposed_premerge;
by a dummy ; /* SORTing the obs by the random number */
RUN;
/** Step 7: Create global macro variable for the number of Frequency bins
(strata) **/
proc sql noprint;
select count(*)
into: strata
from exp_time;
quit;
%let strata=&strata;
%put &strata; /* check macro variable */
/** Step 7: Using a dynamic array randomly assign months between time of
eligibility to proxy exposure to create
a proxy exposure date for the unexposed group
**/
DATA final_unexposed;
merge trans_merge_exp unexposed_premerge;
```

```
by dummy;
b=_n_; /* creating a var that corresponds to observation number
for ease of qa*/
array c[*] c_timetcntc1 - c_timetcntc&strata;
array k[*] cutpt1 - cutpt&strata;
if b <= k[1] then add_months = c[1];
else if b GT k[dim(k) - 1] then add_months = c[dim(k)];
else
do _n_ = 2 to dim(k) - 1;
if k[_n_ - 1] LT b LE k[_n_] then do;
add_months = c[_n_];
leave;
end;
end;
exposure_date=elig_date+(add_months*30);/* create
proxy control start date from
case distribution of time to
contact */
exposure_month=month(exposure_date);
TimeToContc=intck('month',elig_date,exposure_date);
format elig_date exposure_date date9.;
*drop cutpt1--cutpt&strata c_timetcntc1--c_timetcntc&strata;
RUN;
/** Step 9: Compare Frequency distribution of time between eligibility and
exposure (timetoc) and
exposure dates between exposed and unexposed
**/
PROC FREQ DATA=final_unexposed;
table timetocontc;
ods output onewayFREQs=unexposed_check;
```

```
RUN;
PROC SORT DATA=unexposed_check out=unexpcheck(keep=TimeToContc Percent
rename=(Percent=co_pct));
by timetocontc;
RUN;
PROC SORT DATA=exp_time out=expcheck(keep=TimeToContc Percent
rename=(Percent=Ca_pct));
by timetocontc;
RUN;
DATA QA_TIMETOC;
merge expcheck unexpcheck;
by timetocontc;
pct_diff=ca_pct-co_pct;
format pct_diff 8.1;
RUN;
PROC SORT DATA=exposed_edate out=exp_edate (keep=f_exposure_date percent
rename=(percent=ca_pct));
by f_exposure_date;
RUN;
PROC FREQ DATA=final_unexposed;
format exposure_date MONYY.;
table exposure_date;
ods output onewayFREQs=unexposed_edate;
RUN;
PROC SORT DATA=unexposed_edate out=unexp_edate(keep=f_exposure_date percent
rename=(percent=co_pct));
by f_exposure_date;
RUN;
DATA QA_event_date;
merge exp_edate unexp_edate;
```

```
by f_exposure_date;
pct_diff=ca_pct-co_pct;
format pct_diff 8.1;
RUN;
```

Reference: R. Harvey, D.D. Jankus, D. Mosley, Random assignment of proxy event dates to unexposed individuals in observational studies: An automated technique using SASp, UnitedHealthcare, MWSUG (2012).
